# Identification and Comparison of Aroma-Active Compounds in Different Chinese Dark Teas

**DOI:** 10.3390/molecules31152562

**Published:** 2026-07-23

**Authors:** Huai-Jin Xiang, Mou-Ming Zhao

**Affiliations:** 1 School of Biology and Biological Engineering, South China University of Technology, Guangzhou 510641, China; bilov3er@mail.scut.edu.cn; 2 School of Food Science and Engineering, South China University of Technology, Guangzhou 510641, China

**Keywords:** Chinese dark tea, aroma-active compounds, GC-MS, GC-O, sensory

## Abstract

To investigate the influence of manufacturing process on the flavor of Chinese dark tea, the volatile profiles of five varieties (Fuzhuan, Pu-erh, Liubao, Bailiang, and Tianjian) were analyzed using gas chromatography–mass spectrometry/olfactometry (GC-MS/O). A total of 178 volatile compounds were identified by GC-MS, among which linalool and its oxides were the most abundant aroma-active components, particularly in Pu-erh tea, which underwent the longest fermentation. GC-O analysis revealed 11 compounds with relative odor activity values (ROAVs) greater than 1, including *β*-gulonic aldehyde, (E,Z)-2,6-nonadienal, *β*-isopropenyl, (Z)-4-hexenal, 3-methylbutanal, (E,Z)-2,4-decadienal, (E,E)-2,4-decadienal, hexanal, linalool, and (R/Z)-linalool oxide. These were considered the key aroma-active compounds contributing to the overall aroma of dark tea. Principal component analysis further indicated that the distinctive woody and aged notes of Pu-erh tea were associated with linalool, (E/Z)-linalool oxide, 1,2,3-trimethylbenzene, *β*-gulonic aldehyde, benzaldehyde, and citronellol. In contrast, *α/β*-ionone and a series of fatty acid derivatives such as (E,Z)-2,6-nonadienal, (Z)-4-heptenal, (E,Z)-2,4-dodecadienal, (E,E)-2,4-dodecadienal, and hexanal were mainly responsible for sweet, grassy, and herbal notes. These substances are particularly prominent in Fuzhuan and Bailiang teas. Our findings demonstrate that processing techniques significantly affect the aroma quality of dark tea, providing a valuable reference for variety discrimination and process optimization.

## 1. Introduction

Tea is widely consumed all around the world due to its potential health function, mild taste and refreshing fragrance. Depending on raw material and manufacturing process, teas can be divided into four categories: unfermented tea (green tea), semi-fermented tea (oolong tea), full-fermented tea (black tea) and post-fermented tea (dark tea) [1]. Green tea and black tea accounted for the majority of tea consumption in the world, while dark tea was exclusive to China with an increasing yield of 318.9 thousand tons in 2018 [2]. As the concept of a healthy diet becomes increasingly ingrained in people’s minds, dark tea has gained high popularity in China due to its numerous beneficial health functions, including antioxidant, anti-obesity, and hypoglycemic effects. In addition to its health benefits, flavor is another key factor contributing to the popularity of dark tea [3]. The typical characteristics of dark tea are a mellow and slightly bitter taste, along with a pure aroma, often described as “stale,” “fungal,” or “betel nut-like” [4].

**Figure 1:**
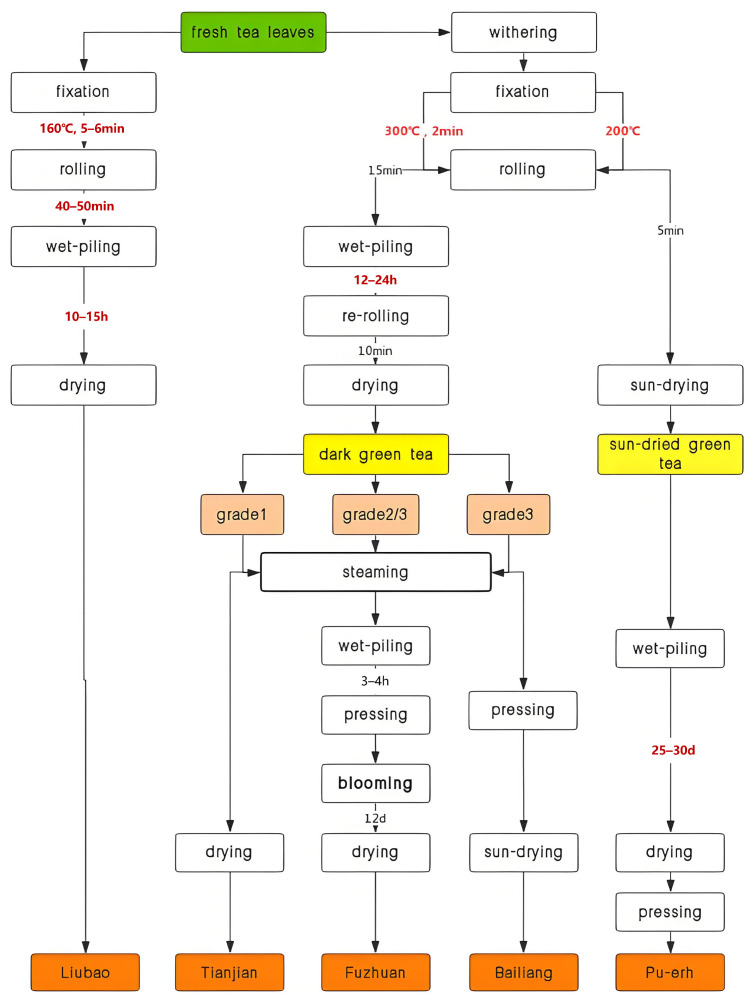
Manufacturing process flowcharts for five representative Chinese dark teas.

The distinctive aroma characteristics of dark tea originate from its manufacturing process, particularly the fermentation stage. The five representative manufacturing processes for dark tea are outlined in Figure 1. The common manufacturing procedure of dark tea includes withering (reducing moisture), fixation (inactivating enzymes), rolling (releasing aroma), wet-piling/post-fermented (microbial action) and drying [5]. The differences can be summarized as follows: for Bailiang tea, the withering process is not included, the fixation temperature is lower than that of the others, and the rolling time is much longer [6]. Pu-erh tea is made from sun-dried green tea, and it has the longest post-fermentation (wet-piling) process (25–30 days) [7]. Tianjian, Fuzhuan and Bailing tea originate from different grades of intermediate product called dark green tea, and the high-grade dark green tea is made from high-tenderness tea leaves. Tianjian is a loose tea, while Fuzhuan and Bailiang are pressed teas (brick-shaped and column-shaped) [8]. Moreover, in the “blooming” process of Fuzhuan tea, the tea brick is inoculated with a special microorganism (*Eurotium cristatum*) to enhance flavor [9].

Microorganisms play an essential part in the manufacturing and flavor development of dark teas. For example, *Aspergillus* and *Penicillium* are the most common microorganisms in dark teas [10]. Zhu et al. found that *Candida* sp., *Aspergillus niger* and *Penicillium* were the major strains responsible for the post-fermentation of dark tea [11]. *Aspergillus niger* was dominant in Pu-erh tea and can promote the formation of monoterpene alcohols, methoxybenzene and derivatives during the long-term piling stage [12]. And *Penicillium* was rich in Liubao tea. In addition, *Eurotium cristatum* (also called “golden flower”) was considered to generate the characteristic “fungal aroma” of Fuzhuan tea by increasing the contents of terpene alcohols and aldehydes [12]. Therefore, the flavor characteristics and aroma composition of different types of dark tea were various. Compared with green tea and black tea, the most distinctive feature of dark tea lies in its fermentation process, and the activity of fermenting microorganisms likely contributes to the formation of richer and more characteristic aroma compounds.

Since 1975, a total of 358 volatile compounds have been detected in teas using gas chromatography–mass spectrometry (GC-MS) [13]. In our laboratory, an in-house dataset of tea flavor compounds was compiled from 60 published manuscripts (Table 1), of which 28 studies focused on green tea and 17 on black tea. However, only 11 publications have addressed dark tea, indicating that the flavor of dark teas requires further research.

As shown in Table 1, a total of 358 aroma-active compounds in teas had been discovered by GC-MS and GC-O, including acids, alcohols, aldehydes, alkanes, aromatic compounds, esters, furans (one), ketones, alkenes, phenols, pyrazines, sulfur compounds and ethers. Ninety-five aroma-active compounds that have been reported over three times are listed in Table 2, and the odorants of dark tea are marked in bold. The majority of these aroma-active compounds are alcohols (62), aldehydes (52), esters (49), ketones (45), aromatic compounds (24) and pyrazines (24). Alcohols (18) and aldehydes (26) were the most abundant classes of aroma-active compounds in teas. In particular, Linalool (19 times), geraniol (16 times), phenyl acetaldehyde (13 times), methional (12 times), β-ionone (12 times), hexanal (12 times), indole (11 times), *β*-damascenone (11 times) and (E,Z)-2,6-nonadienal (11 times) were the most common aroma compounds smelled in the teas. Considering the studies of dark tea, only 29 aroma-active compounds were identified [13] and two types of dark tea were studied, which indicated that the aroma-active compounds in dark tea had not been fully explored.

In this study, volatile and aroma-active compounds of teas were reviewed and listed. Then the aroma-active compounds in five different types of Chinese dark teas (Pu-erh, Bailiang, Fuzhuan, Tianjian, and Liubao) were determined by GC-MS/O and their aroma contributions were estimated by the relative odor activity value (ROAV). Multivariate data analysis on the concentrations of odorants and sensory evaluation was carried out to find the key odorants for each type of dark tea. This result would provide a better understanding of dark tea’s flavor, preliminarily reveal the impression of dark tea processing technology on the formation of its aroma, and meet the increasing demand for high-quality dark tea production.

## 2. Results

### 2.1. Volatiles of Dark Tea

**Figure 2:**
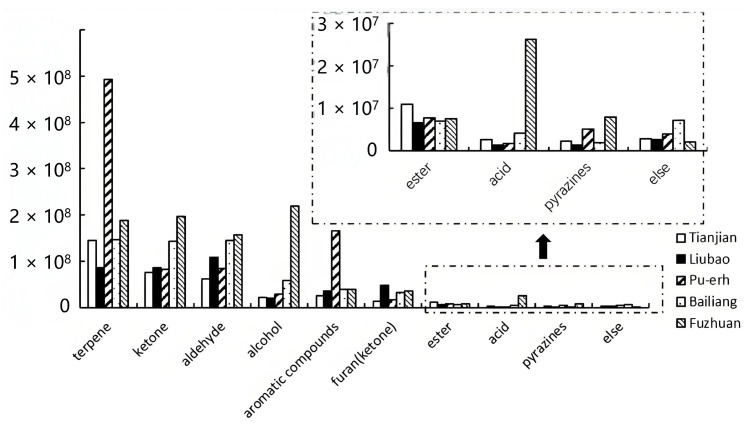
The relative peak area of different chemical classes in Chinese dark tea.

The volatile compounds detected in five Chinese dark teas were listed in Table S1 and the total relative peak area of different chemical classes was shown in Figure 2. A total of 200 volatiles belonging to 10 classes was found, and terpenes (21.77–55.30%), ketones (9.35–24.38%), aldehydes (9.43–27.17%), alcohols (3.27–24.85%) and aromatic compounds (4.54–18.62%) were the major volatile chemicals of the dark tea. The volatile profiles of Pu-erh tea and Fuzhuan tea were obviously different from other dark teas, i.e., Pu-erh tea was rich in terpenes and methoxy benzenes, while Fuzhuan tea contained higher contents of alcohols and acids, which could contribute to their unique fermentation flavor.

A total of 200 volatile substances were detected (Table S1). After further identification, it was determined that 178 of these substances were aroma components, 21 were pollutants or interfering substances, and one was an unknown substance. Several compounds detected in this study require critical evaluation regarding their origin, as they may represent laboratory contaminants, analytical artifacts, or column/background impurities rather than genuine tea constituents. For example, 1,2-benzenedicarboxylic acid bis(2-methylpropyl) ester (diisobutyl phthalate, DIBP), a common plasticizer leached from plasticware, tubing, or solvent contamination; fluorene and phenanthrene, polycyclic aromatic hydrocarbons (PAHs) which are not typical tea constituents and likely originate from column contamination or solvent impurities; and 2,4-di-tert-butyl-phenol and 3,5-di-tert-butyl-4-hydroxybenzaldehyde, which are synthetic anti-oxidants commonly used as stabilizers in solvents and plastic materials. In addition, caffeine was detected in the volatile fraction but it is a non-volatile alkaline compound. Its presence in the volatile fraction might be attributed to co-extraction during the 2 h SDE process or carry-over contamination. Edulan I is an isolation artifact produced from the reactive hemiacetals and is rarely found in natural plants. This substance might have been produced as a result of thermal degradation, oxidation and rearrangement reactions that occurred during the SDE treatment of the sample. Although the GC-MS results were influenced by the SDE process, the main aroma compounds identified in this study were consistent with those reported in previous tea studies that employed milder extraction methods (e.g., HS-SPME and SAFE), suggesting that the overall conclusions remain robust [14]. These compounds are listed in Table S1 for completeness. The presence of these pollutants underscores the importance of adhering to the correct operating procedures, including appropriate preprocessing methods, regular column cleaning, and the use of glassware over plasticware whenever possible.

**Figure 3:**
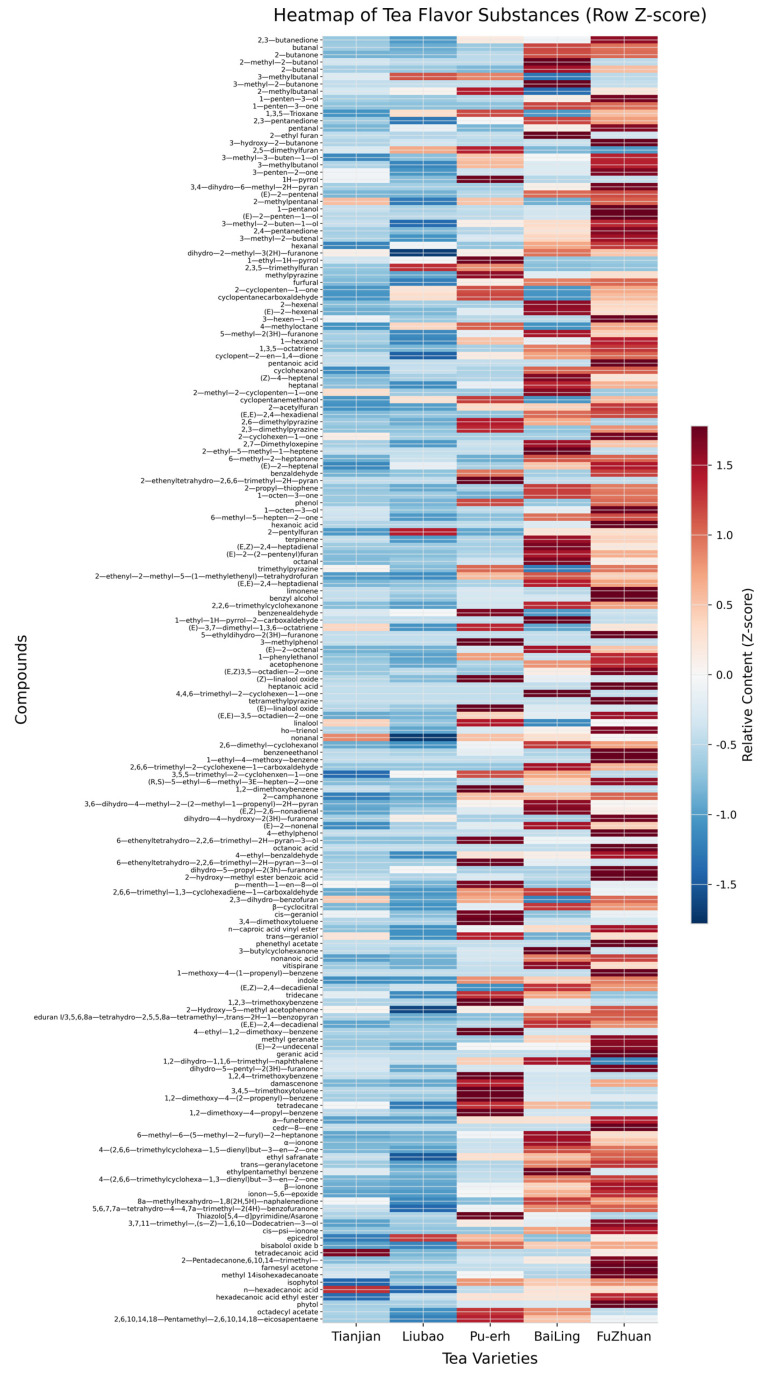
Volatile compounds of Chinese dark tea detected by GC-MS (to mitigate the marked discrepancies in absolute concentrations among different compounds, the raw data were standardized row-wise (i.e., per compound) prior to plotting. The color scale on the right denotes the Z-score: red indicates that the relative content of a compound in a given sample exceeds the overall mean, with darker shades reflecting greater enrichment; conversely, blue indicates a relative content below the overall mean, with darker shades reflecting greater scarcity. White represents values approximating the mean).

The main aroma components of the five types of tea are shown in Figure 3. As shown in Figure 3, the five dark teas all had 127 volatiles in common, which accounted for 67.55% of the total volatiles. Among these compounds, the contents of linalool and its oxides (2,3-butanedione, hexanal and (E,E)-2,4-heptadienal) were much more abundant. In particular, linalool and its oxides ranged from 15% (in Fuzhuan tea) to 48.2% (in Pu-erh tea). Linalool and its oxides are responsible for the sweet and flowery notes of teas and are mainly derived from glycoside precursors by enzymatic hydrolysis [14]. It has been reported that monoterpenes such as linalool increase remarkably from sun-dried green tea to Pu-erh tea [15]. That might indicate that the difference in linalool content of these dark teas is mainly due to the differences in the post-fermentation processes. 2,3-butanedione (4.58–10.41%) was one of the high-content compounds found in five dark tea samples, and it has also been detected in green tea and black tea, and the higher concentration of 2,3-butanedione in the former was considered to be a key factor for the difference in the aromas of green tea and black tea [16]. Hexanal (0.91–5.63%) and (E,E)-2,4-heptadienal (0.67–5.23%) were reported to contribute green and fatty notes to black tea aroma [17]. In addition, Pu-erh tea was found to be rich in 1,2,3-trimethoxybenzene and 1,2,4-trimethoxybenzene; these methoxybenzene compounds have previously been identified as key aroma-active compounds in Pu-erh tea [18].

### 2.2. Aroma-Active Compounds in Dark Tea

GC-O analysis was employed to screen for aroma-active compounds in dark tea, and 43 odorants were identified, comprising 13 aldehydes, seven ketones, five terpenes, three aromatic compounds, four alcohols, and nine unidentified compounds (Table 3). Most of these compounds have been reported as aroma-active compounds in previous studies (Table 1). (E,Z)-2,4-decadienal was first reported as an aroma-active compound in tea products. (E,Z)-2,4-Decadienal, described as having corn chip, stale, and hay notes, was found to have a positive effect on the aroma of fresh tomato and cheese [19]. The retention indices of (E,Z)-2,4-decadienal and its isomer (E,E)-2,4-decadienal were similar to those seen in the study of Lazari et al. [20,21].

Relative odor activity values (ROAVs) of aroma-active compounds were calculated to estimate their contributions to the aroma of dark tea (Table 3). *β*-Damascenone exhibited the highest ratio of peak area percentage to odor threshold in the Tianjian, Fuzhuan, and Pu-erh samples and was therefore designated as the most significant aroma-active compound (ROAV = 100). This value served as a reference for calculating the ROAVs of the other odorants. (E,Z)-2,6-Nonadienal was the odorant with the highest ROAV in the Bailing and Liubao teas. Both (E,Z)-2,6-nonadienal and β-damascenone were common aroma-active compounds in teas, having been reported in over 11 out of 25 publications (Table 2). *β*-Damascenone, in particular, has been reported to contribute substantially to the aromas of green tea, black tea, and oolong tea, owing to its extremely low odor threshold (0.004 μg/L) [26]. (E,Z)-2,6-Nonadienal has a unique cucumber-like flavor and is responsible for the green notes of green tea and black tea [27,28]. Eleven other compounds—namely, β-ionone (woody, sweet), (Z)-4-heptenal (green, potato), 3-methylbutanal (malty), (E,Z)-2,4-decadienal (green, fatty), (E,E)-2,4-decadienal (green, fatty), hexanal (green, grassy), linalool (floral, sweet), and (E/Z)-linalool oxide (woody)—all with ROAVs > 1, were considered to be the key aroma-active compounds in dark tea.

Aroma-active compounds in tea are generated from four main pathways: carotenoid derivatives, lipid derivatives, glycoside derivatives, and the Maillard reaction [29]. Aromas originating from carotenoids mainly depend on enzymatic or non-enzymatic reactions, such as thermal degradation or oxidation under acidic conditions [30,31]. *β*-Damascenone and *β*-ionone are two representative carotenoid-derived aromas. The relative peak areas of *β*-damascenone and *β*-ionone in Pu-erh and Bailing teas were higher than those in the other tea samples, which may be attributed to the long-term fermentation (enzymatic reaction) of Pu-erh tea and the long-term sun-drying process (thermal degradation) of Bailing tea [32]. Lipid-derived aromas were higher in Fuzhuan and Bailing teas. These aromas are formed via two main pathways: lipoxygenase-mediated lipid oxidation and oxidation reactions initiated by free radicals, such as thermal oxidation and photo-oxidation [33]. However, since the enzymes from tea leaves are inactivated by the fixation process, the lipid-derived aromas of dark tea are likely generated by microbial activities, as well as by thermal oxidation or photo-oxidation during the manufacturing process [34].

For example, Wang et al. found that aldehyde compounds such as (E)-2-pentenal, (E)-2-hexenal, 1-penten-3-ol, (E,E)-2,4-heptadienal, and (E,Z)-2,4-heptadienal increased significantly after the fermentation process [35]. Some monoterpene alcohols (linalool, geraniol) and aromatic alcohols (2-phenylethanol) exist in tea leaves in the form of glycosides, which are considered to undergo hydrolysis during the fermentation process of dark tea [36]. Consistent with these previous studies, higher contents of linalool, linalool oxides, and geraniol were detected in Pu-erh tea. Additionally, 1,2,3-trimethoxybenzene (stale) is another characteristic aroma compound originating from gallic acid through fungal methylation in Pu-erh tea [37]. In the present experiment, this compound was detected in all five dark teas, but was only perceived by sensory evaluation in Pu-erh tea.

### 2.3. Sensory Evaluation and PCA Analysis

**Figure 4:**
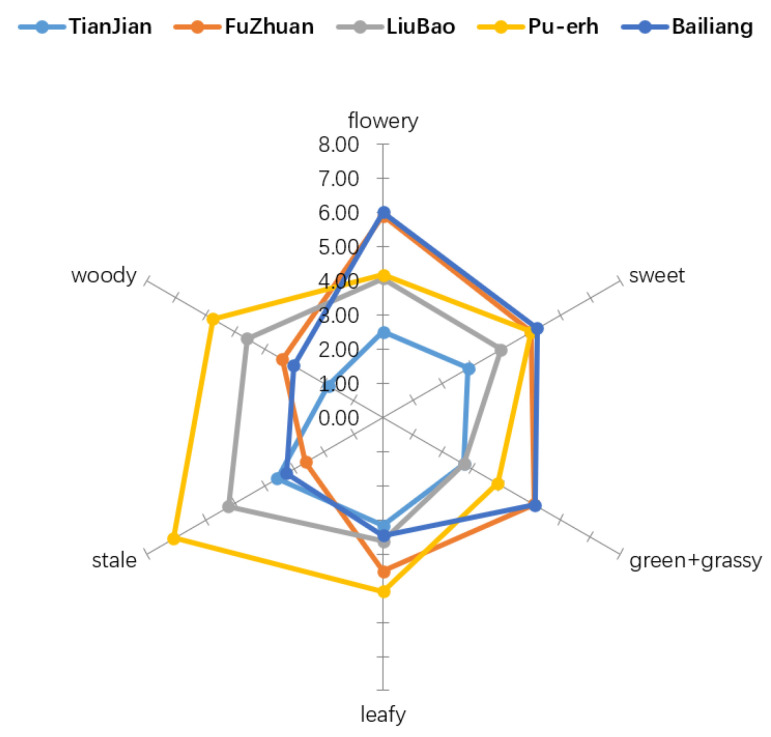
Odor sensory profiles of dark teas.

**Figure 5:**
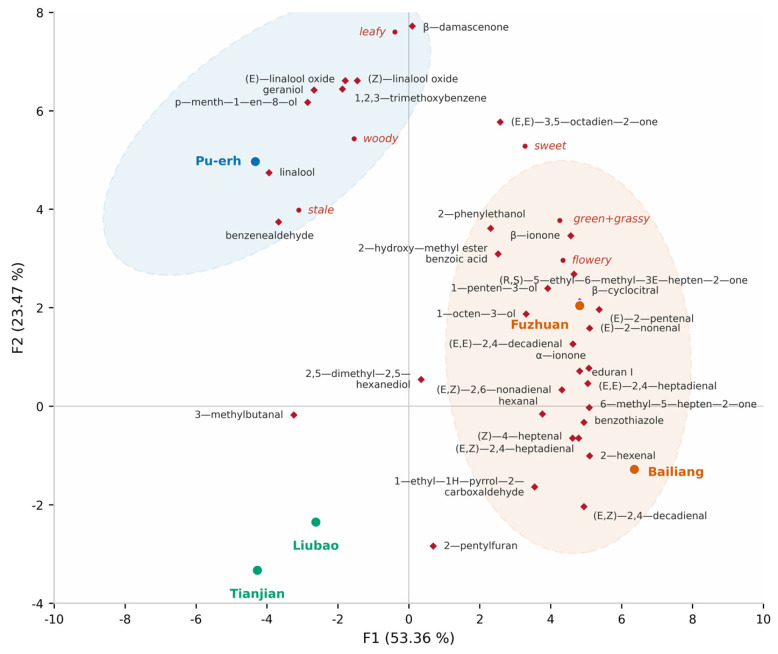
Principal components analysis performed on aroma-active compounds of dark tea.

To improve the characterization of dark tea types and their odor notes, sensory evaluation and principal component analysis (PCA) were carried out. The sensory evaluation results for the five dark teas are shown in Figure 4, and the PCA results of aroma-active compounds and sensory attributes are displayed in Figure 5. As shown in Figure 4, Pu-erh and Liubao teas were rich in woody and stale odors, while Fuzhuan and Bailing teas were characterized by floral and green notes. The aroma profile of Tianjian tea was the weakest, which may be because Tianjian tea is mainly made from more tender tea leaves; consequently, its aroma content may not be as rich as that of the other dark tea samples. Moreover, Fuzhuan and Pu-erh teas also exhibited a distinct leafy aroma.

As shown in the PCA score scatter plot (Figure 5), PC1 and PC2 accounted for 76.83% of the total variance (53.36% and 23.47%, respectively). The five dark teas were divided into three classes: (i) Pu-erh tea, located in the second quadrant and characterized by stale and woody aromas; (ii) Fuzhuan tea and Bailing tea, situated in the positive region of PC1 and associated with floral, green, and sweet aromas; and (iii) Tianjian tea and Liubao tea, located in the third quadrant and lacking distinct flavor attributes or aroma-active compounds.

Most of the aldehydes were located in the positive region of PC1, indicating that these aldehydes might be a key factor in characterizing the aroma of Fuzhuan and Bailing teas. The aldehydes detected in the GC-O analysis were mostly described as having “green and fatty” notes, which was consistent with the findings of the existing study summarized above. On the other hand, β-damascenone, linalool, linalool oxides, geraniol, benzaldehyde, and 1,2,3-trimethoxybenzene were associated with stale, woody, and leafy aromas. It has already been confirmed that the strong stale and woody aroma of Pu-erh tea is related to methoxybenzenes, especially 1,2,3-trimethoxybenzene [38]. Although β-damascenone, linalool, geraniol, and benzaldehyde are commonly reported to exhibit floral, sweet, and rose-like notes in GC-O analysis, some studies on tea aroma have also described linalool and geraniol as woody [39]. Furthermore, in research on the aroma of red wine and soy paste, β-damascenone and benzaldehyde have similarly been characterized as woody [40]. These floral-like aromas may contribute to the woody aroma of dark teas. In the sensory evaluation, the leafy note was associated with linalool oxides, and the sweet note with β-damascenone. The PCA analysis yielded the same result: the leafy note correlated well with the two linalool oxides, and the sweet note also showed a strong association with β-damascenone and β-ionone (woody, sweet). 3-Methylbutanal (malty) was the only compound close to Tianjian tea and Liubao tea in the PCA analysis, and its ROAV values in Tianjian and Liubao teas were 5.9 and 12.8, respectively, indicating that it was a particularly important aroma-active compound for these two teas. 3-Methylbutanal is derived from leucine and is a key aroma compound responsible for cocoa flavor in cocoa beans. Tianjian tea and Liubao tea did not show any obvious features in the PCA analysis, which may be because their sensory scores and aroma contents (GC-MS data) were lower than those of the other dark teas; they appeared to be too weak to be clearly characterized in the PCA analysis. However, Liubao tea still revealed a fairly strong woody and stale smell.

## 3. Discussion

The volatile and aroma-active compound analyses revealed distinct flavor profiles among the five Chinese dark teas, which can be primarily attributed to differences in raw materials, processing techniques, and the degree of microbial fermentation. Pu-erh tea exhibited a unique stale and woody aroma, characterized by remarkably high contents of terpenes (up to 55.30%) and methoxybenzenes, particularly 1,2,3-trimethoxybenzene and 1,2,4-trimethoxybenzene. These methoxybenzene compounds, generated from gallic acid through fungal methylation during long-term post-fermentation, have been identified as key contributors to the characteristic stale odor of Pu-erh tea [37,38]. Additionally, the elevated levels of linalool and its oxides (48.2% of the total volatiles) in Pu-erh tea suggest extensive glycoside hydrolysis during its prolonged fermentation process, further distinguishing it from other dark teas.

Fuzhuan and Bailing teas were distinguished by floral, green, and sweet notes, as evidenced by their higher contents of alcohols, aldehydes, and lipid-derived volatiles. Fuzhuan tea contained the highest proportion of alcohols (24.85%), while Bailing tea showed the highest ROAV of (E,Z)-2,6-nonadienal, a cucumber-like aldehyde responsible for green notes. These differences may arise from lipoxygenase-mediated lipid oxidation and free-radical-initiated oxidation (including thermal and photo-oxidation) occurring during the manufacturing process, particularly during the sun-drying step of Bailing tea [33]. The higher aldehyde contents in these two teas, including hexanal and (E,E)-2,4-heptadienal, further support their green and fatty sensory attributes. The floral character of Fuzhuan tea is additionally enhanced by glycoside-derived compounds such as geraniol and linalool released during fermentation. Tianjian and Liubao teas, however, exhibited relatively weaker aroma profiles. This may be attributable to the use of tender raw materials in Tianjian tea, which contain lower concentrations of precursor compounds for volatile formation. Notably, 3-methylbutanal, a Maillard reaction product derived from leucine with a distinct malty and cocoa-like aroma, was the most characteristic aroma-active compound in both Tianjian and Liubao teas (ROAVs of 5.9 and 12.8, respectively), distinguishing them from the other three teas. The absence of prominent sensory features in these two teas, as indicated by their clustering in the third quadrant of the PCA plot, suggests that their moderate fermentation intensity and shorter processing durations limit the generation of potent odor-active volatiles. From a mechanistic perspective, the flavor differentiation among these five dark teas reflects the differential dominance of four aroma formation pathways: carotenoid degradation, lipid oxidation, glycoside hydrolysis, and the Maillard reaction. The high levels of *β*-damascenone and *β*-ionone in Pu-erh and Bailing teas indicate significant carotenoid-derived aroma accumulation through enzymatic and thermal degradation, respectively. Conversely, the prevalence of lipid-derived aldehydes in Fuzhuan and Bailing teas highlights the role of oxidation processes during their unique manufacturing stages. Collectively, these findings demonstrate that the flavor diversity of Chinese dark teas is fundamentally shaped by the interaction between processing parameters (fermentation time, drying method, and raw material maturity) and the corresponding biochemical transformation pathways.

## 4. Materials and Methods

### 4.1. Tea Leaves

Five commercial Chinese dark teas were purchased from Hunan province (Fuzhuan, Tianjian, Bailiang, Xiangtea Co., Ltd., Anhua, China), Guangxi province (Liubao, Shanye Tea Co., Ltd., Nanning, China) and Yunnan province (Pu-erh, Menghai Tea Factory, Dali, China), respectively. All samples were harvested in Spring 2023 and stored in their original packaging in a dry, dark room at 20–25 °C for 2–3 months before analysis.

### 4.2. Chemicals

Geraniol (≥99.0%), phenylacetaldehyde (95%), β-damascenone (AR), hexanal (98%), linalooloxide (≥97.0%), (Z)-4-heptenal (≥98.0%), (−)-linalool (≥95.0%), (E,E)-2,4-decadienal (≥89%), Dimethyl sulfide (≥99%), 4-Hydroxy-2,5-dimethyl-3(2H)-furanone (HDMF, 98%), β-Ionone (≥97%) were purchased from Sigma-Aldrich (Steinheim, Germany). Sodium chloride and dichloromethane were of high commercial grade and purchased from Sinopharm Chemical Reagent Co., Ltd. (Shanghai, China).

### 4.3. Isolation of Volatile Compounds

The volatile compounds were isolated by means of simultaneous distillation extraction (SDE). Fifteen gram of tea leaves, 300 mL of distilled water, 75 g of sodium chloride and 20 μL of internal standard (2-methyl-3-heptanone, 168 ppm, in methanol) were mixed in a 1000 mL three-neck round bottom flask. Fifty milliliter of dichloromethane was added to another flask as an organic extract solvent. Both flasks were connected to a Likens–Nickerson apparatus and heated right up to their boiling points. Distillation–extraction was continued for 2 h. After extraction, the organic phase was dried by adding anhydrous sodium sulfate and kept at −20 °C overnight. The extract was concentrated to approximately 2 mL with a vigreux, and to an exact volume of 0.5 mL under a high-purity nitrogen steam. Before nitrogen concentration, the 2 mL extract was purified with a 0.22 μm organic filter.

### 4.4. GC-MS/GC-O Analysis

GC-MS analysis was conducted on a Ultra GC, equipped with a triplus automated sampler and a quadrupole DSQ II MS (Thermo Finnigan, San Jose, CA, USA). The column type was TR-5ms (30 m × 0.25 nm × 0.25 μm, J&W Scientific, Folsom, CA, USA). Helium was used as carrier gas with a flow rate of 1.0 mL/min. The split ratio was 1:20. The GC heating program was as follows: the temperature of the column was maintained at 40 °C for 2 min, ramped to 120 °C at 4 °C/min, holding for 2 min, and then raised to 280 °C at a rate of 10 °C/min and held at 280 °C for 5 min. The injection port and the ion source temperature were set at 250 °C. The mass spectrometer was operated in electron impact (EI) mode. The ionization energy, detector voltage, scan range and scan rate applied for the analysis were 70 eV, 350 V, *m*/*z* 35–350 and 3.00 scans/s, respectively. Chromatograms and mass spectra were evaluated using Xcalibur™ software Version 2.0 (Thermo Finnigan, San Jose, CA, USA). The identification of volatile compounds was based on mass spectra and retention indices (RIs) in comparison to the data in the literature and the NIST 08 database.

Olfactometry analysis was performed using the strength method under the same conditions of the GC-MS analysis. Eight assessors (4 males, 4 females, aged 22–35) were recruited from South China University of Technology and screened for olfactory sensitivity using the Sniffin’ Sticks test (identification of 16 common odorants). Sample presentation order was fully randomized. Each sample was evaluated by different assessor combinations on different days to minimize order effects. Sniffing sessions were limited to 90 min with a mandatory 15 min break every 30 min. Assessors were instructed to avoid consuming spicy food, coffee, or tea for 30 min prior to analysis. No more than two samples were evaluated per assessor per day. We employed the strength method (not frequency-based consensus). The final odor intensity (OI) was calculated as the arithmetic mean of all valid scores. A compound was deemed aroma-active only if detected by at least 4 out of 8 assessors (≥50% detection frequency). The odor intensity (OI) was scaled ranging from 0 to 3 (1 = week, 2 = medium, 3 = strong).

To estimate the contribution of each aroma-active compound to the overall flavor, the relative odor activity value (ROAV) was applied. It was defined as follows [14]:

$${ROAV}_{x}=100\times \frac{T_{stan}}{T_{x}}\times \frac{{C\%}_{x}}{{C\%}_{stan}}$$

ROAV_x_—relative odor activity value of aroma-active compound x.

T_stan_—odor threshold of the most valuable aroma-active compounds detected in the sample.

T_x_—odor threshold of aroma-active compound x.

C%_stan_—peak area percentage of the most valuable aroma-active compound.

C%_x_—peak area percentage of aroma-active compound x.

### 4.5. Sensory Evaluation

Aroma characteristics of the dark tea samples were evaluated by quantitative descriptive analysis (QDA) according to the method described by Yuan et al. [7]. Eight panelists (four females and four males, aged 20–35 years) were recruited from South China University of Technology and trained using selected standard odorants characteristic of tea aroma. The training program comprised three sessions prior to formal evaluation: (1) descriptor recognition and familiarization; (2) ranking exercises and intensity calibration against a 3-point reference scale (low, medium, high); and (3) practice profiling on commercial dark tea samples, followed by group discussion to harmonize individual scoring. The aroma attributes were defined as follows: flowery (phenylacetaldehyde), sweet (*β*-damascenone), green/grassy (hexanal), leafy (linalool oxide), hay-like ((Z)-4-heptenal), fatty ((E,E)-2,4-decadienal), sulfurous (dimethyl sulfide), caramel-like (HDMF), stale (undefined), and woody (*β*-ionone). Panelists were required to correctly identify each reference standard on two consecutive occasions and to achieve inter-panelist consensus before proceeding to the formal QDA. During the evaluation process, each attribute was scored using a scale from 0 to 9 (the more prominent the aroma characteristics are, the higher the score). Aliquots of 30 mL of each sample were transferred into 50 mL plastic cups labeled with random three-digit codes, capped, and presented for assessment. Duplicate evaluations were conducted for each sample in two separate sessions on non-consecutive days to verify reproducibility.

### 4.6. Statistical Analysis

To mitigate the marked discrepancies in absolute concentrations among different compounds, the raw data of GC-MS were standardized row-wise (i.e., per compound) prior to plotting. Principal component analysis (PCA) was conducted with the relative ion-peak area of the key aroma-active compounds to determine the relationship amongst five kinds of dark tea using XLSTAT software (version 2019.02.02, Addinsoft, Paris, France). All assays were performed in triplicate, and the results were expressed as the means. Means values were separated using Duncan’s multiple range test when ANOVA was statistically significant (*p* < 0.05) (SPSS 22.0, Chicago, IL, USA).

## 5. Conclusions

This study systematically characterized the aroma profiles of five representative Chinese dark teas using GC-MS/O combined with sensory evaluation and multivariate analysis. A total of 178 volatiles and 41 aroma-active compounds were identified, among which 11 key odorants with ROAV > 1 were highlighted, including β-damscenone, (E,Z)-2,6-nonadienal, β-ionone, (Z)-4-heptenal, 3-methylbutanal, (E,Z)-2,4-decadienal, (E,E)-2,4-decadienal, hexanal, lin-alool, and (E/Z)-linalool oxide. Notably, (E,Z)-2,4-decadienal has been reported as an aroma-active compound in dark tea for the first time. PCA and sensory evaluation results indicated that the distinctive woody and stale flavor of Pu-erh tea was associated with linalool, (E/Z)-linalool oxide, 1,2,3-trimethoxybenzene, β-damascenone, benzaldehyde, and geraniol. In contrast, the sweet, green, and grassy odor of Fuzhuan and Bailing teas was primarily attributed to β-ionone and several fatty acid derivatives. Tianjian and Liubao teas were characterized by 3-methylbutanal as their most distinguishing aroma-active compound. These findings significantly advance our understanding of dark tea aroma chemistry by elucidating the association between tea types and their distinctive aroma characteristics, thereby providing critical insights for quality control, authentication, and the targeted development of premium products tailored to consumer preferences. Several limitations of this study should be acknowledged, including the use of SDE, which may introduce thermal artifacts, and the analysis of only a single batch per tea type. Future investigations should therefore employ complementary extraction techniques (e.g., HS-SPME, SAFE) and incorporate multi-batch sampling to further validate the present findings.

## Figures and Tables

**Table 1:** The chemical classes of volatile and aroma-active compounds in teas.

	Green Tea		Oolong Tea		Black Tea		Dark Tea		Others		All
Category	MS ^a^	Olf ^b^	MS ^a^	Olf ^b^	MS ^a^	Olf ^b^	MS ^a^	Olf ^b^	MS ^a^	Olf ^b^	Olf ^b^
Alcohols	55	33	37	31	57	30	30	10	25	11	62
Ketones	45	25	25	19	48	19	20	3	8	7	45
Aldehydes	32	24	40	32	53	27	21	3	21	14	52
Pyrazines	18	17	10	8	8	7	0	-	1	1	24
Ester	35	16	31	22	36	10	18	-	10	7	49
Furans (One)	22	12	6	5	13	7	7	1	8	5	16
Aromatics	27	9	7	4	17	8	31	8	3	3	25
Acids	14	9	5	4	29	9	5	1	7	7	19
Phenols	14	8	1	1	7	4	4	-	10	9	11
Sulfurs	6	6	12	12	7	5	0	-	1	1	20
Others	12	6	1	-	5	2	1	-	1	1	7
Alkenes	32	3	12	4	23	5	17	3	27	2	15
Alkanes	23	2	4	2	20	7	12	-	1	-	11
Ethers	0	-	2	2	1	-	0	-	0	-	2
Pyrans (One)	1	-	0	-	2	-	0	-	1	-	-
Sum	336	170	193	146	326	140	166	29	124	68	358

^a^ Numbers of volatile compounds detected by GC-MS; -, no compounds detected in GC-MS. ^b^ Numbers of aroma-active compounds detected by GC-O; -, no compounds detected in GC-O.

**Table 2:** The summary of aroma-active compounds in teas.

NO.	Compounds	Odor Quality ^a^	Frequency ^b^
Acids (5)			
1	hexanoic acid	green, acid	5
2	phenylacetic acid	sweet	5
3	butanoic acid	buttery, rancid	3
4	2/3-Methyl butanoic acid	sweaty	3
5	3-phenylpropionic acid	animal-like	3
Alcohols (18)			
6	linalool/β-linalool ^c^	floral	19
7	trans-geraniol ^c^	floral	16
8	trans-Linalool oxide (furanoid) ^c^	leafy, citrus	8
9	phenylethyl alcohol ^c^	honey-like	7
10	cis-Linalool oxide (furanoid) ^c^	leafy, citrus	7
11	nerolidol ^c^	floral (rose), apple, green	6
12	nerol ^c^	floral	5
13	maltol	sweet	5
14	cis-Linalool oxide (pyranoid) ^c^	sweet	5
15	1-pentanol ^c^	green	5
16	(Z)-3-hexen-1-ol ^c^	green	5
17	α-Terpineol ^c^	mint	5
18	Benzylalcohol ^c^	fruity	4
19	1-octanol ^c^	waxy, sweet, floral, citrus-like	3
20	2-Penten-1-ol ^c^	fresh green, metallic	3
21	1,8-cineol	mint	3
22	hotrienol ^c^	fresh, hyacinth	3
23	1-penten-3-ol ^c^	green	3
Aldehydes (26)			
24	phenyl acetaldehyde ^c^	honey-like	13
25	hexanal ^c^	green, almond-like	12
26	(E,Z)-2,6-nonadienal	cucumber-like	11
27	(E)-2-hexenal ^c^	green, apple	9
28	3-methylbutanal	malty	7
29	(E,E)-2,4-decadienal	deep-fried, fatty	7
30	(E,E)-2,4-heptadienal ^c^	fatty, nutty	6
31	benzaldehyde ^c^	fragrant, sweet, almond	6
32	heptanal ^c^	fatty, oily, nutty, green	6
33	Octanal	fat, lemon, green	6
34	(E)-2-nonenal ^c^	fatty, green	5
35	(E,E)-2,4-nonadienal ^c^	deep-fried	5
36	(Z)-4-heptenal	biscuit-like	5
37	(Z)-3-hexenal	leaf-like	5
38	2-methylbutanal	green grass, malt, almond	5
39	furfural	baked bread, almond	5
40	pentanal ^c^	woody, vanilla, nutty	4
41	decanal ^c^	orange, waxy	4
42	nonanal ^c^	fat, citrus, green	4
43	(E)-2-heptenal	soap, fat	4
44	5-methyl-2-furfural	burnt	4
45	2-methylpropanal	pungent, malt, green	4
46	trans-4,5-epoxy-(E)-2-decenal	sweet, juicy, metallic	4
47	(E,E)-2,4-hexadienal	green	3
48	(E)-2-pentenal ^c^	pungent, green, apple, tomato	3
49	(E)-2-octenal ^c^	green, nut, fatty	3
Aromatic Compounds (3)			
50	indole ^c^	animal-like	11
51	vanillin	vanilla-like	9
52	1,2-Benzopyrone ^c^	sweet, camphor	7
Esters (4)			
53	(Z)-methyljasmonate ^c^	floral	8
54	methyl salicylate ^c^	floral, green	7
55	methyl anthranilate	citrus, fruity	5
56	ethyl acetate	pineapple	3
Furan [One] (6)			
57	4-hydroxy-2,5-dimethyl-3(2H)-furanone	caramel-like	9
58	3-hydroxy-4,5-dimethyl-2(5H)-furanone	seasoning-like	8
59	5-decanolide	coconut-like	7
60	4-nonanolide ^c^	sweet	6
61	dihydroactinidiolide ^c^	sweet, woody, floral	5
62	2-Pentyl-furan ^c^	buttery, green bean-like	3
Ketones (13)			
63	β-ionone ^c^	woody, violet-like	12
64	β-damascenone	boiled, apple-like	11
65	(Z)-1,5-octadien-3-one	geranium-like, metallic	9
66	2,3-butanedione	buttery	8
67	1-octen-3-one	mushroom-like	8
68	jasminlactone	sweet	7
69	3-methyl-2,4-nonanedione	strawy, fruity	7
70	cis-jasmone ^c^	green	7
71	α-Ionone ^c^	woody, berry, floral, nutty	5
72	geranyl acetone ^c^	hay-like	4
73	6-methyl-5-hepten-2-one ^c^	herbaceous, pungent, oily	3
74	o-aminoacetophenone	grape-like	3
75	2,3-pentanedione	cream, butter	3
Phenols (5)			
76	2-methoxyphenol	burnt	10
77	2-methoxy-4-vinylphenol	spicy	7
78	eugenol	spicy	7
79	β-Cresol	phenolic	6
80	(E)-isoeugenol	floral, spicy	3
Pyrazines (9)			
81	2-ethyl-3,5-dimethylpyrazine	nutty	8
82	2,5-dimethylpyrazine	scorched rice	7
83	2-Ethylpyrazine	nutty	5
84	2,3,5-trimethylpyrazine	nutty	5
85	2-ethyl-3,6-dimethylpyrazine	roasty, sweet	4
86	2,3-diethyl-5-methylpyrazine	roasty, earthy	4
87	2-ethyl-5-methylpyrazine	sweet, roasted, nutty	3
88	2-ethyl-6-methylpyrazine	roasty, sweet	3
89	2-isobutyl-3-methoxypyrazine	earthy, musty	3
Sulfur Compounds (4)			
90	methional	potato-like	12
91	4-mercapto-4-methyl-2-pentanone	meaty	3
92	dimethyl sulfide	cabbage, sulfur, corn, molasses	3
93	benzothiazole	gasoline, rubber	3
Others (2)			
94	2-acetyl-1-pyrroline	roasty	5
95	2-Acetyl pyrrole	roasted, smoke	4

^a^ Odor description perceived at the GC sniffing port. ^b^ Number of times reported in studies of tea. ^c^ Aroma-active compounds that had been detected in dark tea.

**Table 3:** Aroma-active compounds of Chinese dark tea identified by GC-O.

Compounds	Description ^a^	Tianjian		Fuzhuan		Liubao		Pu-erh		Bailiang		Threshold (ppb) ^d^
		Oi ^b^	RO AV ^c^	Oi ^b^	RO AV ^c^	Oi ^b^	RO AV ^c^	Oi ^b^	RO AV ^c^	Oi ^b^	RO AV ^c^	
ketone												
β-damascenone	flower, fruity	2.8	100	2	100	3	97.1	3	100	3	50.3	0.004 ^e^
α-ionone	woody, sweet	2	0.1	1.2	0.1	-	0.1	-	0.0	2	0.3	5 ^e^
(E,E)-3,5-octadien-2-one	fruity	1.2	1.0	-	2.0	1	1.4	1.5	0.9	1.3	0.8	0.5 ^e^
β-ionone	woody, sweet	1.5	7.3	1.2	9.0	2	7.9	1	3.2	-	6.0	0.2 ^e^
6-methyl-5-hepten-2-one	green, grassy	2.7	0.1	3	0.1	3	0.1	2.5	0.0	3	0.1	50 ^f^
(R,S)-5-ethyl-6-methyl-3E-hepten-2-one	sweet	-	-	-	-	-	-	3	-	3	-	-
2-pentylfuran	green, grassy	-	0.3	-	0.5	2	3.5	-	0.1	-	0.4	6 ^e^
aldehyde												
(Z)-4-heptenal	green, potato	2.5	7.3	3	7.2	3	13.9	3	2.4	3	14.9	0.06 ^e^
(E,Z)-2,4-decadienal	green, fatty	-	0.0	-	0.0	1.5	0.0	1.5	0.0	2.5	0.0	10 ^e^
(E,E)-2,4-decadienal	green, fatty	2.5	0.0	2	0.0	2	0.0	1.5	0.0	3	0.0	180 ^e^
3-methylbutanal	malty	1.8	5.9	2	1.3	2	12.8	2	1.9	2	0.6	0.33 ^f^
benzaldehyde	flower	3	1.2	1.8	0.3	3	1.7	3	0.5	1	0.2	6.3 ^e^
(E,Z)-2,6-nonadienal	cucumber-like	2	39.5	2.5	45.9	2.5	100	2.5	25.2	2.8	100	0.003 ^e^
hexanal	green, grassy	1.5	13.5	1.5	14.3	2.5	39.8	1	4.3	2.5	12.1	0.39 ^f^
(E,E)-2,4-heptadienal	green, fatty	-	0.3	0.8	0.5	1	0.4	1	0.1	1.5	0.7	11.91 ^f^
β-cyclocitral	green, fruity	1.5	0.1	2	0.1	1.5	0.0	1	0.0	-	0.1	5 ^e^
(E)-2-pentenal	green, grassy	-	0.0	-	0.1	1.5	0.0	1	0.0	-	0.1	17.22 ^f^
2-hexenal	malty	-	0.0	2	0.0	3	0.0	2	0.0	-	0.0	58.57 ^f^
(E,Z)-2,4-heptadienal	green, fatty	-	0.0	-	0.0	1	0.0	1.5	0.0	1	0.0	10,000 ^e^
(E)-2-nonenal	green, fatty	2.2	0.0	-	1.9	2.5	2.9	-	0.8	2.7	3.2	0.08 ^g^
terpene												
(Z)-linalool oxide	woody	0.8	4.4	1	1.7	1.5	1.6	1.5	5.8	-	2.3	6 ^h^
(E)-linalool oxide	woody	-	2.9	1	1.2	2.5	1.9	1	4.8	1.8	1.4	6 ^h^
linalool	flower, sweet	2	4.7	3	1.0	2.5	3.0	2.8	1.3	2.5	0.4	6 ^e^
geraniol	lemon	2.5	0.6	2	0.2	3	0.3	3	0.3	1.5	0.1	3.2 ^e^
p-menth-1-en-8-ol	fruity	-	-	-	-	1.2	-	1	-	-	-	-
alcohol												
1-octen-3-ol	mushroom	1.5	1.3	2	0.9	1.5	1.2	2	0.2	2	0.4	1 ^e^
1-penten-3-ol	malty	1.5	0.0	1.5	0.1	1.1	0.0	1	0.0	2	0.0	400 ^e^
2-phenylethanol	flower, honey-like	1.8	0.0	2	0.0	2.8	0.0	1	0.0	-	0.0	1000 ^e^
aromatic compounds												
1,2,3-trimethoxybenzene	stale	-	-	-	-	-	-	2.5	-	-	-	-
1-ethyl-1H-pyrrol-2-carboxaldehyde	chocolate	-	-	-	-	-	-	2	-	-	-	-
2-hydroxy-methyl ester benzoic acid	fruity, sweet	-	-	2	-	-	-	1	-	2	-	-
else												
unknown 1	flower	-	-	2	-	-	-	-	-	-	-	-
unknown 2	matcha-like	2.3	-	2.8	-	2.5	-	2	-	3	-	-
unknown 3	matcha-like	2.5	-	3	-	1.5	-	3	-	2.8	-	-
unknown 4	stale, tea-like	3	-	-	-	1	-	1	-	1	-	-
unknown 5	drug-like	0.7	-	2	-	-	-	1	-	0.5	-	-
unknown 6	sweet, fruity	2.5	-	3	-	1.5	-	3	-	-	-	-
Unknown 7	roasted potato	2.2	-	2.8	-	2	-	2.8	-	2.2	-	-
Unknown 8	chocolate	2.8	-	-	-	2	-	2.5	-	1	-	-
Unknown 9	popcorn-like	2	-	-	-	1	-	3	-	-	-	-

^a^ Odor description perceived at the GC sniffing port. ^b^ Odor intensity perceived at the GC sniffing port; -, odor intensity was not perceived. ^c^ Relative odor activity value. ^d^ Odor threshold in water taken from reference; -, odor threshold was unavailable. ^e^ Odor threshold taken from Magagna et al. [22]. ^f^ Odor threshold taken from Zhu et al. [23]. ^g^ Odor threshold taken from Zhu et al. [24]. ^h^ Odor threshold taken from Chen et al. [25]. The odor intensity (OI) was scaled ranging from 0 to 3 (1 = week, 2 = medium, 3 = strong).

## Data Availability

The original contributions presented in this study are included in the article/Supplementary Materials. Further inquiries can be directed to the corresponding author.

## Supplementary Materials

The following supporting information can be downloaded at: https://www.mdpi.com/article/10.3390/molecules31152562/s1, Table S1: Volatile compounds of Chinese dark tea detected by GC-MS.
