# Supplementary material for: Identification and Comparison of Aroma-Active Compounds in Different Chinese Dark Teas
Source: Molecules. 2026 Jul 23;31(15):2562. doi: 10.3390/molecules31152562 (PMC13468435; doi:10.3390/molecules31152562)
Supplement: Supplementary file 1 [file molecules-31-02562-s001.zip › molecules-4335003-supplementary.pdf]

Table S1. Volatile compounds of Chinese dark tea detected by GC-MS.

NO.	RI[a]	Category	Compounds	Relative peak area[b]				
				Tianjian	Liubao	Pu-erh	BaiLing	FuZhuan
1	<600		2,3-butanedione	16.125	12.040	26.963	23.935	44.768
2	<600		butanal	0.532	0.478	0.527	0.923	0.875
3	<600		2-butanone	3.471	3.500	4.122	7.119	6.829
4	626		2-methyl-2-butanol	0.098	-	-	1.938	-
5	637		2-butenal	0.381	0.368	0.340	0.793	0.608
6	643		3-methylbutanal	1.219	2.182	2.009	0.480	1.049
7	644		3-methyl-2-butanone	-	-	-	1.690	-
8	650		cyclohexane	3.269	15.311	24.975	-	16.480
9	651		2-methylbutanal	2.316	2.637	3.834	1.390	2.755
10	677		1-penten-3-ol	4.658	3.412	6.890	20.474	57.357
11	680		1-penten-3-one	3.003	2.908	2.988	16.856	15.328
12	685		1,3,5-Trioxane	-	25.769	41.927	0.645	30.386
13	697		2,3-pentanedione	0.145	0.099	0.189	0.275	0.243
14	699		pentanal	4.420	5.411	4.422	5.729	7.948
15	700		2-ethyl furan	0.974	0.799	1.006	6.011	1.255
16	708		3-hydroxy-2-butanone	0.189	1.057	0.337	0.815	10.487
17	712		2,5-dimethylfuran	1.454	3.101	4.316	0.527	0.198
18	728		3-methyl-3-buten-1-ol	-	0.045	0.182	0.127	0.276
19	732		3-methylbutanol	0.140	0.082	0.246	0.170	0.322
20	735		3-penten-2-one	0.971	0.512	0.833	0.880	1.763
21	745		1H-pyrrol	1.357	0.479	3.471	0.816	0.914
22	748		3,4-dihydro-6-methyl-2H-pyran	-	-	-	0.042	0.137

23	750	(E)-2-pentenal	0.447	0.410	0.880	2.995	3.227
24	756	2-methylpentanal	0.185	0.073	0.182	0.098	0.216
25	761	toluene	0.394	0.350	0.464	1.205	0.643
26	764	1-pentanol	0.661	0.336	0.576	0.855	4.975
27	765	(E)-2-penten-1-ol	0.273	0.237	0.425	1.344	14.698
28	773	3-methyl-2-buten-1-ol	0.089	-	0.137	0.158	0.250
29	776	2,4-pentanedione	0.038	0.024	0.045	0.109	0.217
30	783	3-methyl-2-butenal	0.136	0.035	0.192	0.331	0.561
31	800	hexanal	3.300	7.990	5.328	10.990	13.349
32	806	dihydro-2-methyl-3(2H)-furanone	0.242	0.064	0.241	0.328	0.281
33	809	1-ethyl-1H-pyrrol	0.437	0.635	1.768	0.205	0.228
34	812	2,3,5-trimethylfuran	0.022	0.160	0.127	0.016	0.019
35	825	methylpyrazine	0.094	0.061	0.469	0.177	0.262
36	828	furfural	2.163	0.669	5.152	7.612	8.007
37	829	2-cyclopenten-1-one	-	0.369	0.635	-	0.495
38	838	cyclopentanecarboxaldehyde	-	2.784	4.543	-	3.264
39	842	2-hexenal	0.031	0.045	0.043	0.243	0.133
40	850	(E)-2-hexenal	1.301	1.463	2.725	6.721	3.960
41	852	3-hexen-1-ol	0.228	0.046	0.098	0.098	0.808
42	855	ethylbenzene	0.157	1.120	1.706	0.174	1.295
43	858	4-methyloctane	-	0.376	0.552	-	0.451
44	863	5-methyl-2(3H)-furanone	0.097	0.051	0.135	0.273	0.185
45	865	o-xylene	0.236	1.696	2.433	0.035	1.838
46	867	1-hexanol	0.326	0.249	0.489	0.395	0.614
47	874	1,3,5-octatriene	0.067	0.058	0.095	0.332	0.388
48	876	cyclopent-2-en-1,4-dione	0.169	0.042	0.245	0.317	0.354

49	880	pentanoic acid	-	-	-	-	1.488
50	884	cyclohexanol	-	0.113	0.089	0.343	0.353
51	886	ethenylbenzene	0.242	2.391	3.789	0.180	2.705
52	887	1,4-dimethyl-benzene	-	-	-	-	-
53	896	(Z)-4-heptenal	0.275	0.429	0.458	2.074	1.033
54	898	heptanal	0.752	0.608	0.992	1.587	1.291
55	902	2-methyl-2-cyclopenten-1-one	0.087	-	-	0.209	-
56	903	cyclopentanemethanol	-	0.160	0.278	-	0.196
57	906	2-acetylfuran	0.036	0.073	0.543	0.575	0.870
58	907	(E,E)-2,4-hexadienal	0.046	0.061	0.057	0.318	0.346
59	913	2,6-dimethylpyrazine	0.324	0.207	1.390	0.999	0.374
60	917	2,3-dimethylpyrazine	-	-	0.430	-	0.318
61	927	2-cyclohexen-1-one	0.160	-	-	-	0.502
62	928	2,7-Dimethyloxepine	0.052	0.024	0.066	0.197	0.129
63	940	2-ethyl-5-methyl-1-heptene	-	-	-	2.001	-
64	949	6-methyl-2-heptanone	-	0.073	-	0.316	0.300
65	951	(E)-2-heptenal	0.295	0.453	0.379	0.553	0.699
66	955	benzaldehyde	1.779	1.989	4.946	2.156	5.415
67	965	2-ethenyltetrahydro-2,6,6-trimethyl-2H-pyran	-	-	0.505	-	-
68	967	3-methyl-2(5H)-furanone	-	-	-	-	-
69	968	Aniline	0.381	0.200	0.285	0.659	0.811
70	969	2-propyl-thiophene	0.158	0.058	0.220	1.165	1.003
71	971	1-octen-3-one	0.141	0.143	0.119	0.362	0.329
72	974	phenol	0.119	0.044	1.012	0.106	0.927
73	975	1-octen-3-ol	0.827	0.610	0.758	0.958	2.149
74	980	6-methyl-5-hepten-2-one	4.329	2.620	3.344	8.212	8.758

75	981	hexanoic acid	0.140	0.131	0.465	1.276	8.885
76	984	2-pentylfuran	1.121	10.955	1.499	6.212	6.586
77	988	terpinene	0.141	0.045	0.123	0.468	0.280
78	990	(E,Z)-2,4-heptadienal	1.098	1.125	1.227	8.822	3.922
79	993	(E)-2-(2-pentenyl)furan	0.085	0.091	0.169	0.919	0.617
80	997	octanal	0.128	0.155	0.221	0.687	0.336
81	998	trimethylpyrazine	0.643	0.232	1.148	-	1.106
82	999	2-ethenyl-2-methyl-5-(1-methylethenyl)-tetrahydrofuran	0.118	0.068	0.852	1.093	0.780
83	1005	(E,E)-2,4-heptadienal	2.119	2.597	3.959	18.563	13.501
84	1023	limonene	0.644	0.428	0.877	0.541	4.382
85	1027	benzyl alcohol	0.526	0.142	0.728	0.709	3.775
86	1027	2,2,6-trimethylcyclohexanone	0.385	0.300	0.539	1.215	0.972
87	1035	benzenealdehyde	4.830	5.661	10.268	2.820	4.493
88	1039	1-ethyl-1H-pyrrol-2-carboxaldehyde	-	-	-	1.762	-
89	1040	(E)-3,7-dimethyl-1,3,6-octatriene	0.595	0.170	0.880	0.206	0.541
90	1044	5-ethyldihydro-2(3H)-furanone	-	-	-	-	0.437
91	1046	3-methylphenol	-	-	0.283	-	-
92	1051	(E)-2-octenal	0.439	0.435	0.784	1.924	1.307
93	1053	1-phenylethanol	0.065	0.041	0.214	0.094	0.275
94	1057	acetophenone	0.899	0.799	0.935	1.500	1.723
95	1062	(E,Z)3,5-octadien-2-one	-	0.083	-	0.320	0.933
96	1063	(Z)-linalool oxide	16.416	4.918	112.659	31.703	23.736
97	1069	heptanoic acid	-	-	-	-	1.011
98	1069	4,4,6-trimethyl-2-cyclohexen-1-one	-	-	-	0.272	-
99	1077	tetramethylpyrazine	-	-	-	-	1.857
100	1079	(E)-linalool oxide	10.993	5.998	92.988	20.081	17.594

101	1085	(E,E)-3,5-octadien-2-one	0.304	0.360	1.442	0.912	2.413
102	1092	linalool	17.783	9.269	25.473	5.976	14.677
103	1095	ho-trienol	1.952	0.327	2.931	4.296	10.789
104	1096	nonanal	0.933	0.335	0.860	0.801	0.744
105	1100	2,6-dimethyl-cyclohexanol	0.678	0.315	2.294	4.870	3.852
106	1102	benzeneethanol	0.804	0.143	3.058	0.287	16.944
107	1104	1-ethyl-4-methoxy-benzene	-	-	-	-	0.211
108	1109	2,6,6-trimethyl-2-cyclohexene-1-carboxaldehyde	-	-	-	0.409	0.246
109	1112	3,5,5-trimethyl-2-cyclohenxen-1-one	0.090	1.107	1.951	1.619	0.768
110	1132	(R,S)-5-ethyl-6-methyl-3E-hepten-2-one	-	0.005	0.040	0.148	0.296
111	1135	1,2-dimethoxybenzene	1.340	0.314	14.000	2.069	1.291
112	1138	2-camphanone	-	0.045	0.248	0.253	0.317
113	1143	3,6-dihydro-4-methyl-2-(2-methyl-1-propenyl)-2H-pyran	0.242	0.284	0.656	1.310	0.641
114	1143	(E,Z)-2,6-nonadienal	0.074	0.154	0.243	0.696	0.328
115	1146	dihydro-4-hydroxy-2(3H)-furanone	-	0.981	-	-	3.130
116	1150	(Z)-2-nonenal	-	-	-	-	-
117	1151	(E)-2-nonenal	-	0.118	0.205	0.594	0.356
118	1156	4-ethylphenol	-	-	-	-	1.709
119	1161	6-ethenyltetrahydro-2,2,6-trimethyl-2H-pyran-3-ol	0.512	0.430	11.548	3.506	3.105
120	1164	octanoic acid	-	-	-	-	1.432
121	1165	4-ethyl-benzaldehyde	0.100	0.052	0.200	0.186	0.328
122	1166	6-ethenyltetrahydro-2,2,6-trimethyl-2H-pyran-3-ol	1.803	1.392	41.157	8.674	5.429
123	1173	naphthalene	1.873	3.436	2.734	0.782	0.716
124	1174	dihydro-5-propyl-2(3h)-furanone	-	0.668	-	-	2.738
125	1182	2-hydroxy-methyl ester benzoic acid	0.300	0.153	0.549	0.244	3.731
126	1186	p-menth-1-en-8-ol	10.233	4.348	22.183	5.320	9.101

127	1189	2,6,6-trimethyl-1,3-cyclohexadiene-1-carboxaldehyde	0.916	0.690	3.946	4.744	2.377
128	1207	2,3-dihydro-benzofuran	0.969	0.139	1.096	-	1.282
129	1209	β-cyclocitral	0.196	0.115	0.387	0.849	0.698
130	1213	benzothiazole	0.631	0.431	0.617	0.682	0.694
131	1215	cis-geraniol	1.206	0.507	3.100	0.540	1.193
132	1225	3,4-dimethoxytoluene	0.312	0.248	4.927	0.572	0.728
133	1229	n-caproic acid vinyl ester	0.083	0.047	0.147	0.190	0.302
134	1240	trans-geraniol	5.581	1.670	8.942	2.334	5.781
135	1242	phenethyl acetate	-	-	-	-	1.121
136	1246	edulan Ⅱ	0.088	-	-	0.342	0.326
137	1251	3-butylcyclohexanone	0.097	0.057	0.144	0.751	0.120
138	1257	nonanoic acid	-	0.035	0.178	0.518	0.606
139	1268	vitispirane	0.388	0.175	0.664	3.058	1.607
140	1274	1-methoxy-4-(1-propenyl)-benzene	-	-	-	-	0.018
141	1278	indole	0.106	0.124	1.198	0.980	1.265
142	1282	(E,Z)-2,4-decadienal	0.170	0.243	-	0.814	0.643
143	1290	tridecane	0.722	0.381	1.262	1.046	0.536
144	1292	mint furanone /UNKNOW	-	-	-	0.146	0.135
145	1295	1,2,3-trimethoxybenzene	0.233	0.510	38.585	5.451	2.161
146	1297	2-Hydroxy-5-methyl acetophenone	0.378	0.059	0.391	0.446	0.581
147	1298	eduran Ⅰ/3,5,6,8a-tetrahydro-2,5,5,8a-tetramethyl-,trans-2H-1-benzopyran	0.291	0.061	0.149	1.447	1.451
148	1306	(E,E)-2,4-decadienal	0.502	0.750	0.978	2.964	2.434
149	1307	4-ethyl-1,2-dimethoxy-benzene	0.482	0.399	5.299	0.983	1.051
150	1309	methyl geranate	-	-	-	0.034	0.072
151	1328	(E)-2-undecenal	0.148	0.073	0.361	0.353	0.873

152	1341	geranic acid	-	-	-	-	0.919
153	1346	1,2-dihydro-1,1,6-trimethyl-naphthalene	0.051	0.045	0.100	0.165	-
154	1351	dihydro-5-pentyl-2(3H)-furanone	0.248	0.066	0.238	0.277	0.837
155	1361	1,2,4-trimethoxybenzene	0.506	0.046	13.446	1.086	0.305
156	1371	damascenone	0.250	0.200	1.285	0.467	0.954
157	1375	methyl cinnamate	-	-	-	-	-
158	1387	3,4,5-trimethoxytoluene	0.169	0.157	5.759	0.675	0.313
159	1389	1,2-dimethoxy-4-(2-propenyl)-benzene	0.037	0.025	0.435	0.096	0.105
160	1391	tetradecane	0.821	0.370	1.289	1.029	0.595
161	1393	1,2-dimethoxy-4-propyl-benzene	0.220	0.132	1.273	0.286	0.305
162	1397	a-funebrene	-	-	0.098	0.096	0.187
163	1404	cedr-8-ene	-	-	-	-	0.081
164	1405	6-methyl-6-(5-methyl-2-furyl)-2-heptanone	0.224	0.243	0.460	0.893	0.565
165	1410	α-ionone	0.315	0.316	0.770	2.924	1.783
166	1415	4-(2,6,6-trimethylcyclohexa-1,5-dienyl)but-3-en-2-one	0.447	0.290	0.849	2.405	2.204
167	1426	ethyl safranate	0.159	-	0.262	0.303	0.376
168	1437	trans-geranylacetone	0.801	0.469	0.894	2.250	2.647
169	1450	2,6-Di(t-buty)-4-hydroxy-4-methyl-2,5-cyclohexadien-1-one	0.468	1.087	0.776	1.101	0.503
170	1455	ethylpentamethyl benzene	0.395	0.100	0.281	1.414	0.384
171	1466	4-(2,6,6-trimethylcyclohexa-1,3-dienyl)but-3-en-2-one	0.205	0.163	0.378	1.042	1.186
172	1468	β-ionone	0.909	0.818	2.025	2.777	4.301
173	1471	ionon-5,6-epoxide	0.909	0.818	2.025	2.777	3.620
174	1493	2,4-di-tert-butyl-phenol	0.531	0.637	0.519	0.550	1.898
175	1502	8a-methylhexahydro-1,8(2H,5H)-naphalenedione	1.120	0.568	0.757	1.746	1.500

176	1513	5,6,7,7a-tetrahydro-4-4,7a-trimethyl-2(4H)-benzofuranone	1.893	0.417	2.369	3.783	4.141
177	1533	Thiazolo[5,4-d]pyrimidine/Asarone	-	-	0.384	0.074	-
178	1548	3,7,11-trimethyl-,|s-(Z)|-1,6,10-Dodecatrien-3-ol.	-	0.117	0.422	0.283	0.980
179	1568	cis-psi-ionone	-	-	-	0.240	0.356
180	1571	fluorene	0.107	0.130	0.334	0.102	0.128
181	1598	epicedrol	0.028	0.761	0.563	0.170	0.434
182	1643	bisabolol oxide b	0.062	0.046	0.209	0.168	0.184
183	1661	3,4-Diethyl-1,1'-biphenyl	0.091	0.074	0.129	0.112	0.077
184	1738	tetradecanoic acid	0.242	0.046	0.071	0.069	0.120
185	1740	3,5-di-tert-buty-4-hydroxybenzaldehyde	0.149	0.114	0.217	0.278	0.170
186	1772	phenanthrene	0.613	0.819	1.836	0.433	0.426
187	1819	2-Pentadecanone,6,10,14-trimethyl-	0.328	0.379	0.656	1.048	2.199
188	1822	caffeine	1.181	0.851	2.256	1.045	2.411
189	1837	1,2-Benzenedicarboxylic acid bis(2-methylpropyl) ester	4.241	1.804	3.441	2.827	1.370
190	1886	farnesyl acetone	-	-	-	-	0.494
191	1898	methyl 14isohexadecanoate	0.212	0.153	0.251	0.186	0.472
192	1920	isophytol	-	0.241	0.715	0.613	0.728
193	1933	n-hexadecanoic acid	0.866	0.280	0.485	0.636	0.650
194	1964	hexadecanoic acid ethyl ester	-	0.080	0.138	0.134	0.230
195	2077	phytol	0.876	1.003	2.078	2.239	10.250
196	2122	lauramide	-	0.357	-	0.171	7.264
197	2136	octadecyl acetate	0.186	0.141	0.353	0.312	0.196
198	2209	9-octadecenamide	-	0.517	0.504	0.151	0.901
199	2222	hexadecanamide	0.405	0.316	0.363	-	0.476
200	2453	2,6,10,14,18-Pentamethyl-2,6,10,14,18-eicosapentaene	0.456	0.196	1.304	0.926	0.684

[a] Retention index on the TR-5ms column.

[b] peak area relative to that of internal standard (20 μL of 168 ppm 2-methyl-3-heptanone in methanol).

-, compound was not detected.
